# Four Eremophilane Sesquiterpenes from the Mangrove Endophytic Fungus *Xylaria* sp. BL321

**DOI:** 10.3390/md10020340

**Published:** 2012-02-06

**Authors:** Yongxiang Song, Jiajian Wang, Hongbo Huang, Lin Ma, Jun Wang, Yucheng Gu, Lan Liu, Yongcheng Lin

**Affiliations:** 1 School of Chemistry and Chemical Engineering, Sun Yat-Sen University, 135 Xingang West Road, Guangzhou 510275, China; Email: songxiang0517@126.com (Y.S.); kakinvono@gmail.com (J.W.); huanghb@scsio.ac.cn (H.H.); cesmal@mail.sysu.edu.cn (L.M.); 2 CAS Key Laboratory of Marine Bio-resources Sustainable Utilization, Guangdong Key Laboratory of Marine Materia Medica, RNAM Center for Marine Microbiology, South China Sea Institute of Oceanology, Chinese Academy of Sciences, 164 West Xingang Rd., Guangzhou 510301, China; 3 Guangdong Province Key Laboratory of Functional Molecules in Oceanic Microorganism (Sun Yat-Sen University), Bureau of Education of Guangdong, Guangzhou, 510275, China; Email: wjun@mail.sysu.edu.cn; 4 School of Pharmaceutical Sciences, Sun Yat-Sen University, Guangzhou 510080, China; 5 Syngenta Jealott’s Hill International Research Centre, Bracknell, Berkshire, RG42 6EY, UK; Email: yucheng.gu@syngenta.com

**Keywords:** marine fungus, *Xylaria* sp., mangrove, sesquiterpenes, α-glucosidase

## Abstract

Three new eremophilane sesquiterpenes (**1**–**3**) were isolated from the mangrove endophytic fungus *Xylaria* sp. BL321 together with 07H239-A (**4**), a known analogue of the new compounds. The structures of these compounds were elucidated by analysis of their MS, 1D and 2D NMR spectroscopic data. Compound **4** showed activation activity on α-glucosidase at 0.15 μM (146%), and then, **4** gradually produced inhibitory activity on α-glucosidase with increasing concentration, and the IC_50_ value is 6.54 μM.

## 1. Introduction

Many structurally novel and biologically active metabolites have been isolated and characterized from the fungal genus *Xylaria*, including cytochalasins [1,2,3], phytotoxins [2], xyloketals [4,5,6,7], cyclopeptides [8,9,10], lactones [11,12], xanthones [13,14], sesquiterpenes [15,16], and aromatic derivatives [17,18]. Our previous studies on the mangrove endophytic fungus *Xylaria* sp. 2508 had led to the isolation of a series of the above mentioned compounds [5,6,7,8,9,10,17,18]. These findings on fungus *Xylaria* sp. 2508 encouraged chemical research resulting in the isolation of two new lactones from a endophytic fungus *Xylaria* sp. BL321 [19]. The current study presents additional chemical investigation performed on *Xylaria* sp. BL321 that led to the discovery of three new eremophilane sesquiterpenes (**1**–**3**) and a known analogue 07H239-A (**4**) [15] isolated by extensive silica gel column chromatography and semipreparative HPLC.

Herein, we report the isolation, structure elucidation and enzyme-based biological assays against α-glucosidase of the four eremophilane sesquiterpenes.

## 2. Results and Discussion

Compound **1** was obtained as a white solid. The HREIMS results established the molecular formular C_26_H_34_O_6_ (*m/z* 442.2345 [M]^+^, calcd. 442.2350), and suggested ten degrees of unsaturation in the structure of this compound. Its IR spectrum indicated the presence of a hydroxyl group at 3300~2700 cm^−1^ and carbonyl, together with conjugated carbonyl absorptions at 1728, 1705, 1682 and 1648 cm^−1^. The UV absorption band at 261 nm showed the present of a conjugated dienoate system. The existence of a ketone carbonyl (δ_C_ 197.1, C-8), a formyl group (δ_H_ 9.56, δ_C_ 193.3, C-12), a carboxyl group (δ_C_ 177.6, C-14), and an ester carbonyl (δ_C_ 165.9, C-1') were confirmed by the ^1^H, ^13^C and HMQC NMR data (Table 1). The NMR data also supported the existence of three methyls, seven methylenes including a terminal olefinic carbon (C-13), five unsaturated methines, four aliphatic methines, and three quaternary carbons. Analysis of the COSY spectrum disclosed three spin systems of H-1–H-4, H-6–H-7, and H-2'–H-10' and H-8'–H-11', which led to the establishment of the structure fragments C-1–C-4, C-6–C-7, and the long side chain incorporated a 1,3-diene, respectively. The HMBC correlations of H-4/C-5 and H-6/C-5 suggested that fragments C-1–C-4 and C-6–C-7 were connected through a quaternary carbon (C-5). An isolated methyl (δ_H_ 1.54, δ_C_ 19.8, C-15) was linked to C-5 supported by the HMBC correlations of H_3_-15/C-4, C-5, C-6. The HMBC cross peaks of H-13/C-11, and of H-12/C-11, C-13 indicated that the formyl group (C-12) was attached to a terminal olefin (C-11–C-13) to build the acrylaldehyde moiety. Further HMBC correlations of H-12/C-7 and H-13/C-7 located the unsaturated aldehyde at C-7. The carbonyl (C-8) was conjugated with an olefinic bond (C-9–C-10) to form an α,β-unsaturated ketone, which was proved by the HMBC correlation of H-9/C-8 and H-9/C-10. These HMBC correlations from H-9 to C-1, C-7, C-5, from H_3_-15 to C-10, and from H-6 to C-8, C-10 established the two six-membered rings backbone. This structure elucidation was in agreement with the remaining two degrees of unsaturation. The HMBC correlation between the proton of the oxygen-bearing methine (C-1) and the carbonyl (C-1') implied the presence of an ester bond at C-1. The ^13^C NMR chemical shift (δ_C_ 165.9) of carbonyl carbon indicated it was connected with the 1,3-diene to form a conjugated system as evident by the HMBC correlation of H-3'/C-1'. As determined from the HMBC correlation of H-4/C-14, the carboxyl group was linked to C-4. Thus, the planar structure of compound **1** was established.

**Table 1:** ^1^H and ^13^C NMR data of compounds **1**–**3**, *J* in Hz.

	1 ^a^		2 ^b^		3 ^b^	
Position	δ_C_, mult.	δ_H_ (*J* in Hz)	δ_C_, mult.	δ_H_ (*J* in Hz)	δ_C_, mult.	δ_H_ (*J* in Hz)
1	72.7, CH	5.54, (t, 3.0)	72.7, CH	5.51, (t, 3.0)	73.0, CH	5.53, (bs)
2	30.0, CH_2_	2.17, (m)	29.9, CH	2.16, (m)	29.9, CH_2_	2.19, (m)
		1.75, (m)		1.71, (m)		1.75, (m)
3	20.2, CH_2_	2.36, (m)	20.8, CH_2_	2.31, (m)	20.3, CH_2_	2.32, (m)
		1.87, (m),		1.81, (m)		1.75, (m)
4	53.5, CH	2.47, (dd, 13.2, 3.1)	53.3, CH	2.45, (d, 12.5)	53.6, CH	2.47, (m)
5	38.6, C		38.6, C		38.5, C	
6	43.4, CH_2_	2.30, (m)	43.5, CH_2_	2.29, (m)	43.4, CH_2_	2.31, (m)
		2.14, (m)		2.13, (m)		2.16, (m)
7	43.3, CH	3.75, (dd, 14.7, 4.6)	43.4, CH	3.71, (dd, 14.2, 3.7)	43.4, CH	3.74, (d, 13.0)
8	197.1, C		197.0, C		197.0, C	
9	129.8, CH	6.12, (s)	129.8, CH	6.09, (s)	129.8, CH	6.10, (s)
10	159.1, C		159.2, C		159.3, C	
11	147.9, C		148.0, C		147.9, C	
12	193.3, CH	9.56, (s)	193.3, CH	9.52, (s)	193.3, CH	9.54, (s)
13	136.6, CH_2_	6.37, ( s)	136.6, CH_2_	6.33, (s)	136.6, CH_2_	6.36, (s)
		6.26, (s)		6.22, (s)		6.24, (s)
14	177.6, C		not observed		not observed	
15	19.8, CH_3_	1.54, (s)	19.9, CH_3_	1.24, (s)	19.8, CH_3_	1.56, (s)
1'	165.9, C		166.3, C		167.3, C	
2'	118.6, CH	5.77, (d, 15.2)	114.9, CH	5.72, (d, 15.5)	124.7, C	
3'	146.3, CH	7.28, (ddd, 15.2, 6.4, 3.6)	151.0, CH	7.29, (d, 15.5)	140.1, CH	7.15, (d, 11.3)
4'	128.2, CH	6.20, ( m)	132.7, C		124.2, CH	6.30, (dd, 15.0, 11.3)
5'	146.4, CH	6.18, (m)	144.0, CH	5.91, (t, 7.4)	150.3, CH	5.93, (dd, 15.0, 8.5)
6'	30.9, CH_2_	2.19, (m)	26.8, CH_2_	2.20, (m)	35.6, CH	2.39, (m)
7'	35.6, CH_2_	1.47, (m)	30.0, CH_2_	1.42, (m)	32.2, CH	1.31, (m)
		1.27, (m)		1.22, (m)	44.2, CH_2_	1.35, (m)
8'	34.2, CH_2_	1.36, (m)	34.3, CH	1.33, (m)		1.13, (m)
9'	29.5, CH_2_	1.39, (m)	35.9, CH_2_	1.15, (m)	11.5, CH_3_	0.82, (t, 6.2)
		1.17, (m)				
10'	11.5, CH_3_	0.87, (t, 7.2)	19.2, CH_3_	0.87, (t, 6.2)	12.9, CH_3_	1.94, (s)
11'	19.2, CH_3_	0.89, (d, 6.4)	12.3, CH_3_	1.76, (s)	21.4, CH_3_	1.04, (d, 6.7)
12'			11.5, CH_3_	0.81, (d, 6.3)	19.2, CH_3_	0.81, (3H, d, 7.3)
^a^ Measured in CDCl_3_ at 400 MHz (^1^H) and 100 MHz (^13^C); ^b^ Measured in CDCl_3_ at 500 MHz (^1^H) and 125 MHz (^13^C).						

**Figure 1:**
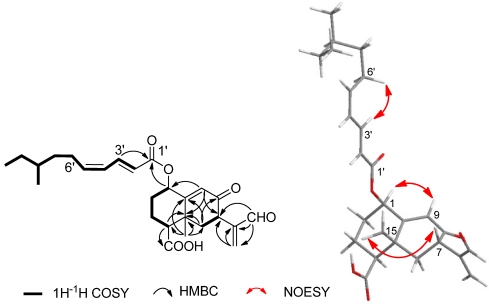
COSY, Key HMBC, and NOESY correlations of compound **1**.

**Figure 2:**
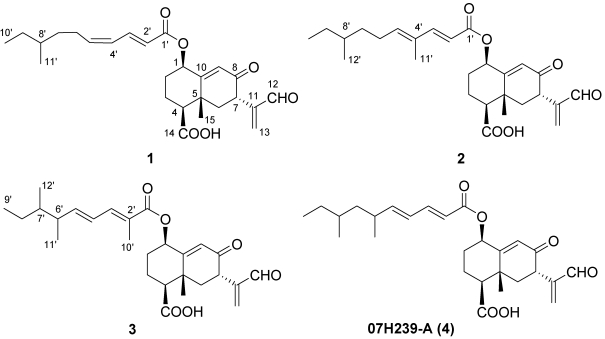
Structure of compounds **1**–**4** isolated fromfungus *Xylaria* sp. BL321.

The relative configuration of **1** was determined by ^3^*J*_H__-H_ values in ^1^H NMR spectrum and NOESY experiment (Figure 1). The small coupling constant (^3^*J*_H-H_ = 2.6 Hz) of H-1/H-2 indicated that the H-1 was equatorially oriented, which was supported by the correlation of H-1/H-9 in NOESY. The large coupling constants of H-4/H-3 and H-7/H-6 (^3^*J*_H-H_ = 13.2, 14.7 Hz, respectively) suggested H-4 and H-7 have axial orientation. The NOESY correlation between H-7 and H_3_-15 located CH_3_-15 and H-7 at the same side of the molecular surface, which led to the *trans* configuration of H_3_-15/H-4. The strongly coupled protons at the double bond C-2'-C-3' (^3^*J*_H-H_ = 15.2 Hz) defined *E*-configuration to this olefinic bond. Though the coupling constant of H-4'/H-5' was not able to be determined due to the overlapped signals, the *Z*-configuration was assigned to the double bond C-4'–C-5' on the basis of the NOE between H-3' and H-6'. Therefore, compound **1** was identified as a new eremophilane sesquiterpene as shown in Figure 2.

**Figure 3:**
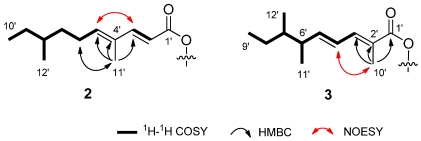
Key COSY, HMBC, and NOESY correlations of the side chain in compounds **2**–**3**.

Compound **2** was obtained as a white solid. Its molecular formula C_27_H_36_O_6_ was established from the peak at *m/z* 455.2446 ([M − H]^−^, calcd. 455.2434) revealed by HRESIMS, which is 14 mass unit higher than **1**. The ^1^H and ^13^C NMR spectroscopic data of **2** disclosed those structural features similar to those found in **1** (Table 1), except the unsaturated proton (δ_H_ 6.20, H-4') signal in **1** was missing, and an unsaturated quaternary carbon signal (δ_C_ 132.7, C-4') and an additional isolated methyl signal (δ_H_ 1.76, s; δ_C_ 12.3, C-11') were observed. Its ^13^C NMR chemical shift value of C-3' shifted downfield from δ_C_ 146.3 in **1** to δ_C_ 150.0 in **2**. Comprehensive comparison of the NMR spectra of **2** with that of **1** revealed that the olefinic proton (H-4') at the side chain of **1** was replaced by a methyl group (C-11') in **2**. This was supported by the COSY correlations of H-5'–H-10', H-12'/H-8' and H-2'/H-3', and the HMBC correlations from H-11' to C-3', C-4', C-5'. (Figure 3) The configuration of the two double bonds were assigned to be *E* configuration due to the large coupling constant (^3^*J*_H-H_ = 15.5 Hz) of H-2'/H-3', as well as the NOE correlations of H-6'/H-11' and H-3'/H-5'. The relative configuration of C-1, C-4, C-5 and C-7 were assigned as the same as those in **1** on the basis of the similar coupling constants. Thus, the structure of **2** was established as shown in Figure 2.

Compound **3** shares the same molecular formula of C_27_H_36_O_6_ with **2**, as determined by the HRESIMS results. The ^1^H and ^13^C NMR data showed the presence of five methyl signals, indicating that **3** has two more methyl groups than **1**. Furthermore, the unsaturated methine (CH-2') and the methylene (CH_2_-6') signals in **1** were absent, while an additional unsaturated quaternary carbon signal at δ_C_ 124.7 (C-2') and an additional methine at δ_H_ 2.39, δ_C_ 35.6 (CH-6') were observed. Comparing the ^1^H and ^13^C NMR spectroscopic data of **3** with those of **1** and **2** revealed that **3** possess an identical double ring backbone to those found in **1** and **2**, but with a different side chain at C-1. The structure of this side chain was deduced from COSY and HMBC spectra. In the HMBC spectrum, the correlations from H_3_-10' to C-1', C-2', C-3' linked the methyl group (Me-10') to quaternary C-2'. The COSY experiment gave a spin system of H-3'–H-9', H-6'–H-11', H-7'–H-12', establishing the remaining structural fragment of the side chain, as shown in Figure 3. The configurations of the two olefins on the side chain were determined to be *E* in the same fashion as that in **2**, based on ^3^*J*_H-4__'*/*H-5__'_ = 15.5 Hz and the NOE of H-10'/H-4'. 

Compound **4** was isolated as the main product, which structure was determined to be the known compound 07H239-A by comparing the 1D and 2D data with the reported data in the literature [15].

The literature suggests that eremophilane sesquiterpenes with an ester side chain possess various biological activities. Compound **4** and its derivatives [20] showed cytotoxicity against cancer cell lines, while eremoxylarins A and B displayed antibacterial activity against *Staphylococcus aureus* and *Pseudomonas aeruginosa* [21], and Sch 420789 had phospholipase D inhibitory activity [22]. Unfortunately, the new eremophilane sesquiterpenes **(1**–**3)** did not show cytotoxic activity against the cell lines of MDA-MB-435, A549, Hep3B, PC-3 in our study, though their structures are highly related to that of **4**. Compounds **1** and **4** were further evaluated for their effects on α-glucosidase using an enzyme-based bioassay. Compound **1** had no detectable effect on α-glucosidase. Compounds **4** showed the best activation effect on α-glucosidase at the concentration of 0.15 μM, which enhanced the enzyme activity to a level of 146%. However, compound **4** showed gradually higher inhibitory activity toward α-glucosidase with increasing concentration, and an IC_50_ value of 6.54 μM. Compound **2** and **3** were not tested due to the limited amounts obtained. 

## 3. Experimental Section

### 3.1. General Experimental Procedures

Melting points were determined on SFW-X-4 apparatus. Optical rotations measurements were carried out on a Polaptronic HNQW5 (Schmidt-Haensch) spectrometer. UV spectra were determined on a UV-2501 PC (Shzmadzu) spectrometer. IR spectra were measured on a 5DX-FTIR (Nicolet) spectrometer. ^1^H NMR and ^13^C NMR data were acquired on a Bruker AVANCE 400 spectrometer at 400 MHz for ^1^H nucleus and 100 MHz for ^13^C nucleus, and a Varian INOVA500NB spectrometer at 500 MHz for ^1^H nucleus and 125 MHz for ^13^C nucleus. TMS was used as an internal standard and chemical shifts (δ) were expressed in ppm. ESIMS, HRESIMS, EIMS and HREIMS were operated on LCQ-DECA-XP (Thermo), LCMS-IT-TOF (Shimadzu), DSQ (Thermo) and Mat95XP (Thermo) mass spectrometers, respectively. Column chromatography (CC) was carried out on silica gel (200–300 mesh, Qingdao Marine Chemical Inc.). HPLC was performed on a 515 pump with a UV 2487 detector (Waters) using an Ultimate XB-C18 column (250 × 10 mm, 5 μ; Welch).

### 3.2. Fermentation

The small agar slice bearing mycelia, which were stocked and incubated at 25 °C on PDA (Potato Dextrose Agar) medium, was placed in GYT medium (1% glucose, 0.1% yeast extract, 0.2% peptone, 0.2% crude sea salt) in 500 mL Erlenmeyer flasks containing 250 mL GYT and incubated at 28 °C, 120 rpm for 6 days as seed culture. A 150 L large scale fermentation was performed using multiple 1000 mL Erlenmeyer flasks, each containing 500 mL GYT. Each flask was inoculated with 1 mL seed culture and incubated at 28 °C for 30 days under static conditions.

### 3.3. Extraction and Isolation

The smashed dry mycelia of the fungus were extracted using EtOAc (EA), and then the extract solution was concentrated to dryness. The EA extract was subjected to silica gel CC eluting with petroleum ether (PE)/EA mixtures of increasing polarity. The filtered fraction (*ca.* 500 mg) eluted by PE/EA (65/35) was further isolated by using extensively semi-preparative RP HPLC eluted with MeOH/H_2_O/acetic acid (85/15/0.1) at a flow rate of 1.0 mL/min, detected under UV 254 nm and afford compounds **1** (41 mg, t_R_ = 32.16), **2** (5 mg, t_R_ = 45.88), **3** (3 mg, t_R_ = 57.15), **4** (340 mg, t_R_ = 38.31), respectively.

Compound **1**: mp 159~161 °C; [α]^25^_D_ −19 (*c* 0.145, MeOH); UV (MeOH) λ_max_ (log ε) 261 (4.58) nm; IR (KBr) *ν*_max_ 3453, 1728, 1705, 1682 and 1648 cm^−1^; ^1^H NMR (CDCl_3_, 400 MHz), ^13^C NMR (CDCl_3_, 100 MHz), see Table 1; EIMS *m/z* (rel. int.) 442 [M]^+^ (0.5), 278 (4), 260 (24), 165 (100), 81 (34). HREIMS *m/z* 442.2345 [M]^+^ (calcd. for C_26_H_34_O_6_, 442.2350).

Compound **2**: mp 141~143 °C; [α]^25^_D_ −14 (*c* 0.047, MeOH); UV (MeOH) λ_max_ (log ε) 262 (4.86) nm; IR (KBr) *ν*_max_ 3366, 1726, 1683, 1647 and 1621 cm^−1^; ^1^H NMR (CDCl_3_, 500 MHz), ^13^C NMR (CDCl_3_, 125 MHz), see Table 1; EIMS *m/z* (rel. int.): 278 (3), 260 (48), 91 (100). HRESIMS *m/z* 455.2446 [M − H]^−^ (calcd. for C_27_H_36_O_6_, 455.2434).

Compound **3**: [α]^25^_D_ −47 (*c* 0.052, MeOH); UV (MeOH) λ_max_ (log ε) 269 (4.06) nm; IR (KBr) *ν*_max_ 3394, 1732, 1704, 1680 and 1651 cm^−1^; ^1^H NMR (CDCl_3_, 500 MHz), ^13^C NMR (CDCl_3_, 125 MHz), see Table 1; EIMS, *m/z* (rel. int.): 278 (1), 260 (98), 111 (100), 69 (52). HRESIMS *m/z* 455.2441 [M − H]^−^ C_27_H_36_O_6_ (calcd. for 455.2434). 

### 3.4. Biological Assays

The enzyme-based assay against α-glucosidase was operated using a modified method based on those previously reported [23,24]. Briefly, α-glucosidase activity was assayed with 1 mL reaction volume in 0.01 M phosphate buffer solution (PBS) at pH 7.0, while *p*-nitrophenyl-α-D-glucopyranoside (PNPG) was used as substrate. 10 μL of enzyme solution (5 U/mL in PBS), 950 μL PBS and 20 μL the tested compound (solution in DMSO) were mixed and incubated at 37 °C for 20 min, and then the substrate PNPG 20 μL (3 mg/mL) was added to initiate the enzyme reaction. The absorbance was immediately determined at 400 nm with spectrophotometer.

## 4. Conclusions

This investigation has led to the isolation of four eremophilane sesquiterpenes including the known compound 07H239-A (**4**), which enriched this family of compounds. Compounds **1**–**3** were found to be inactive to the test cell lines, which supported that their side chain may play an important role in the cytotoxic activity [20]. The activity of compound **4** against α-glucosidase was found for the first time. 

## Supplementary Files

Supplementary File 1: PDF-Document (PDF, 614 KB)
