# Supplementary material for: Four Eremophilane Sesquiterpenes from the Mangrove Endophytic Fungus Xylaria sp. BL321
Source: Mar Drugs. 2012 Feb 6;10(2):340–8. doi: 10.3390/md10020340 (PMC3297001; doi:10.3390/md10020340)

Supplementary Materials

**Supporting Information**	**Page**
S1. EIMS of compound **1**	3
S2. ^{1}H NMR (400 MHz, $CDCl_3$) spectrum of compound **1**	3
S3. ^{13}C NMR (100 MHz, $CDCl_3$) spectrum of compound **1**	4
S4. DEPT and ^{13}C NMR (100 MHz, $CDCl_3$) spectrum of compound **1**	4
S5. HMQC spectrum of compound **1** in $CDCl_3$	5
S6. H-H COSY spectrum of compound **1** in $CDCl_3$	5
S7. HMBC spectrum of compound **1** in $CDCl_3$	6
S8. NOE spectrum of compound **1** in $CDCl_3$	6
S9. EIMS of compound **2**	7
S10. ^{1}H NMR (500 MHz, $CDCl_3$) spectrum of compound **2**	7
S11. ^{13}C NMR (125 MHz, $CDCl_3$) spectrum of compound **2**	8
S12. HMQC spectrum of compound 2 in $CDCl_3$	8
S13. H-H COSY spectrum of compound **2** in $CDCl_3$	9
S14. HMBC spectrum of compound **2** in $CDCl_3$	9
S15. NOE spectrum of compound **2** in $CDCl_3$	10
S16. EIMS of compound **3**	11
S17. ^{1}H NMR (500 MHz, $CDCl_3$) spectrum of compound **3**	11
S18. ^{13}C NMR (125 MHz, $CDCl_3$) spectrum of compound **3**	12
S19. HMQC spectrum of compound **3** in $CDCl_3$	12
S20. H-H COSY spectrum of compound **3** in $CDCl_3$	13
S21. HMBC spectrum of compound **3** in $CDCl_3$	13
S22. NOE spectrum of compound **3** in $CDCl_3$	14

S1. EIMS of compound **1**

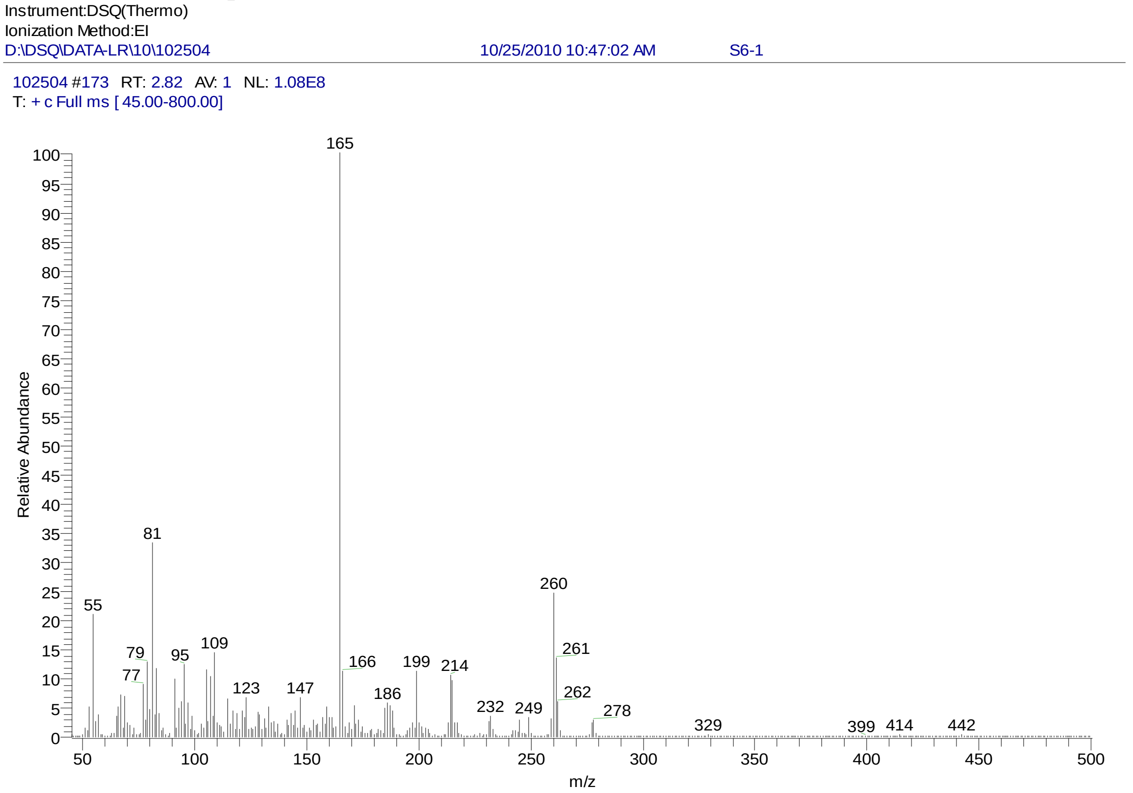

S2. ^{1}H NMR (400 MHz, $CDCl_3$) spectrum of compound **1**

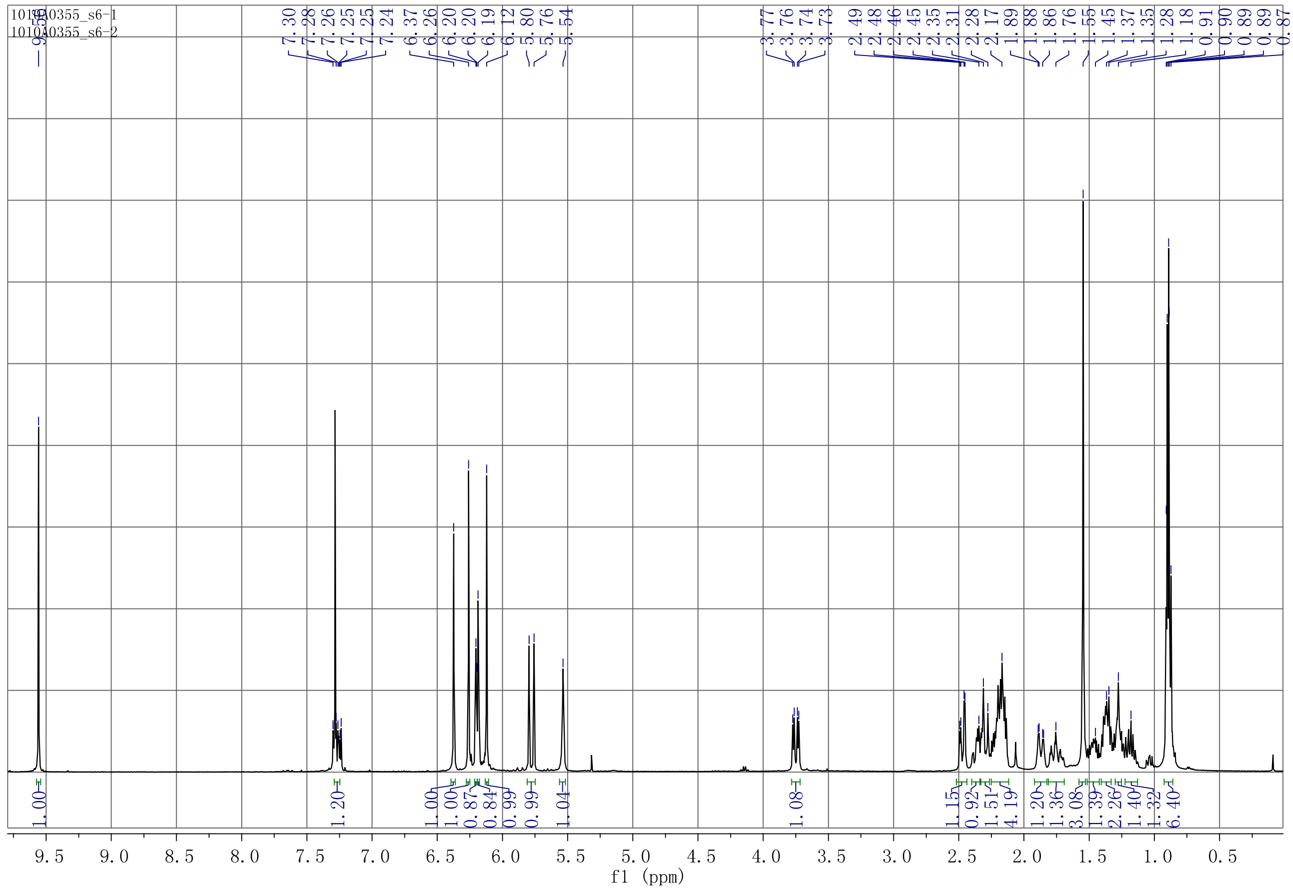

S3. ^{13}C NMR (100 MHz, $CDCl_3$) spectrum of compound **1**

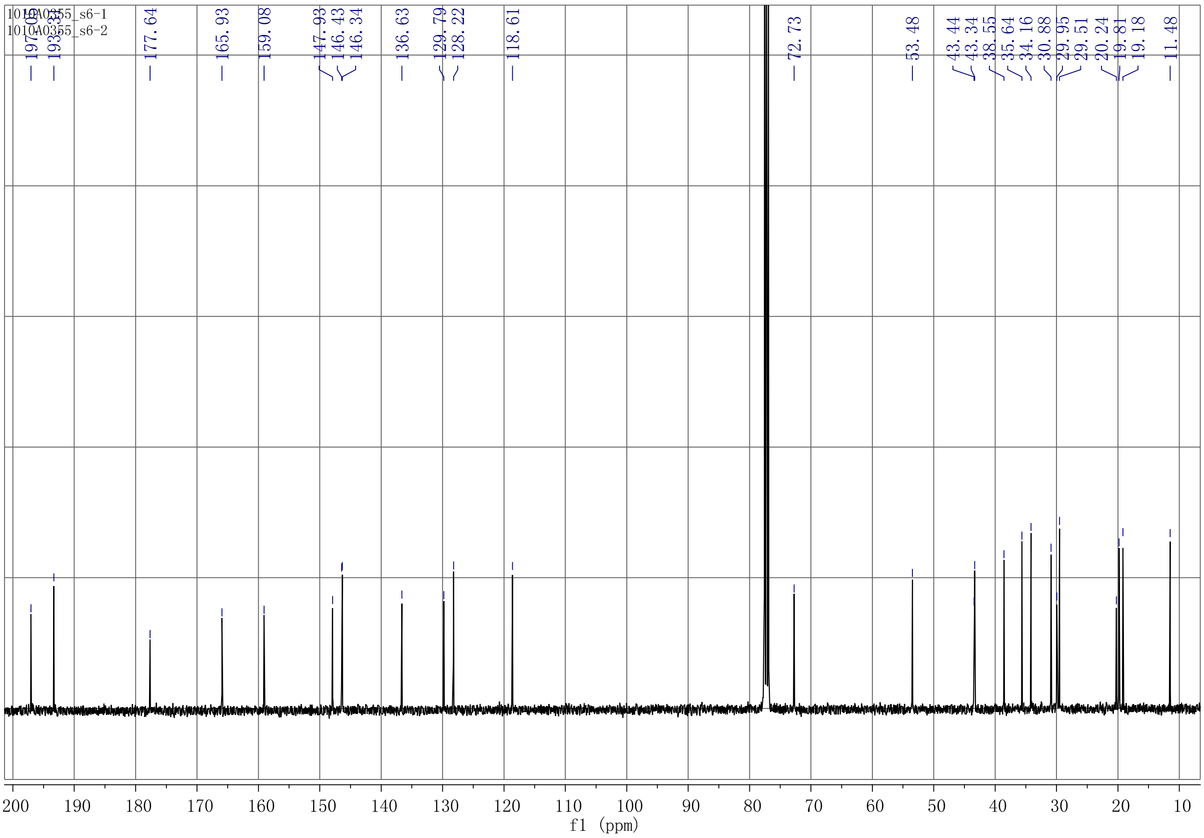

S4. DEPT and ^{13}C NMR (100 MHz, $CDCl_3$) spectrum of compound **1**

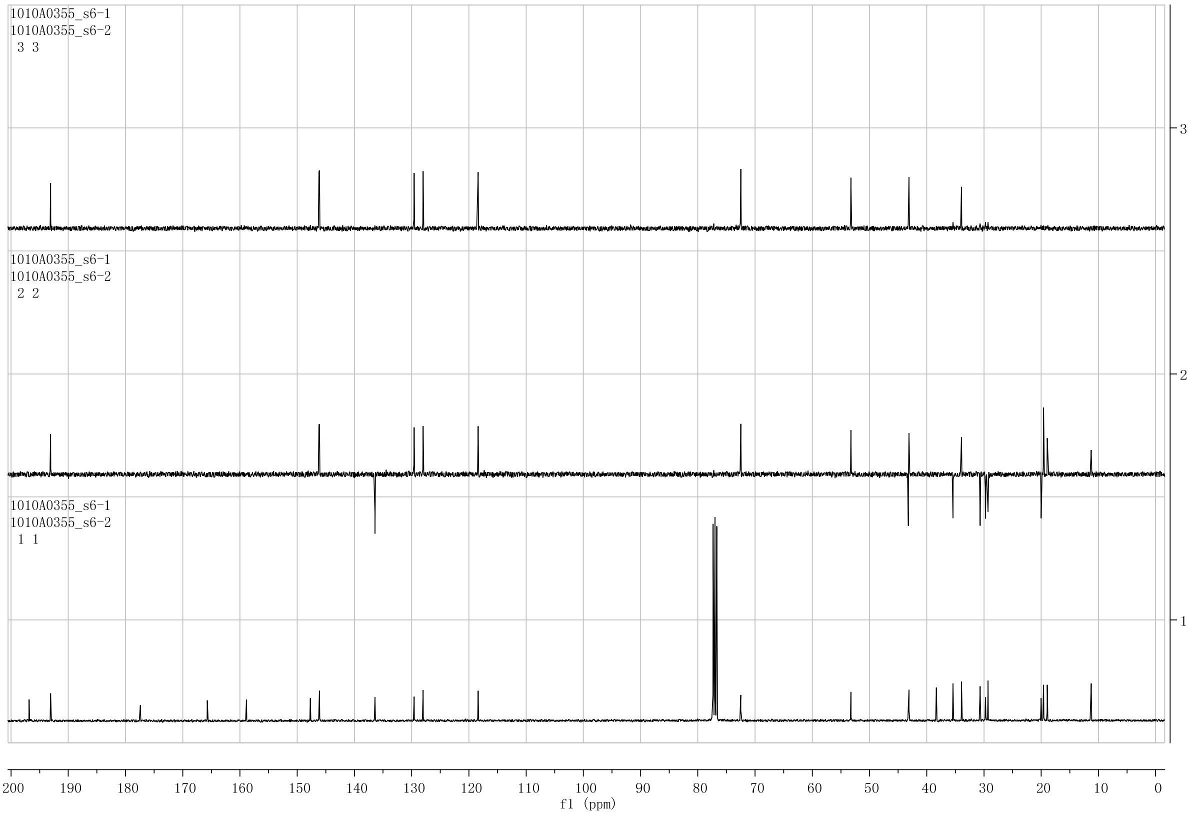

S5. HMQC spectrum of compound **1** in $CDCl_3$

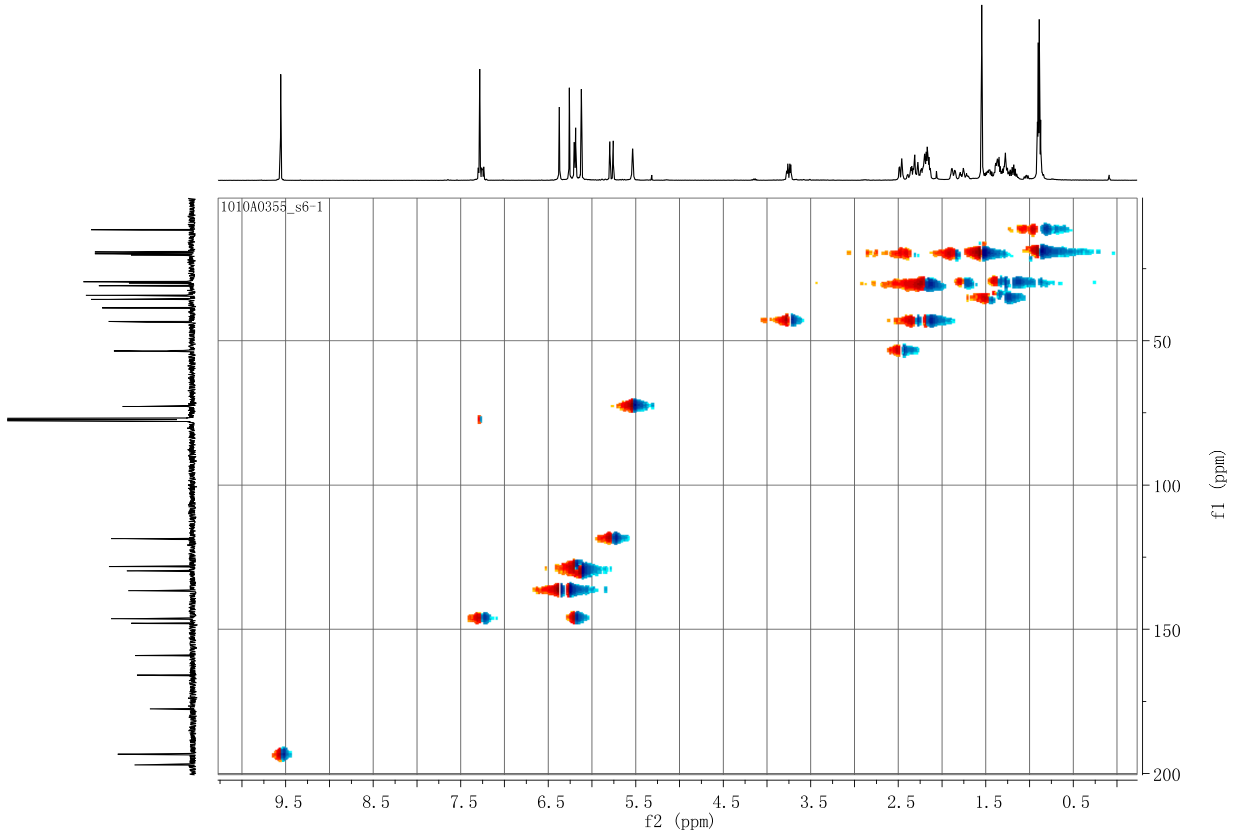

S6. H-H COSY spectrum of compound **1** in $CDCl_3$

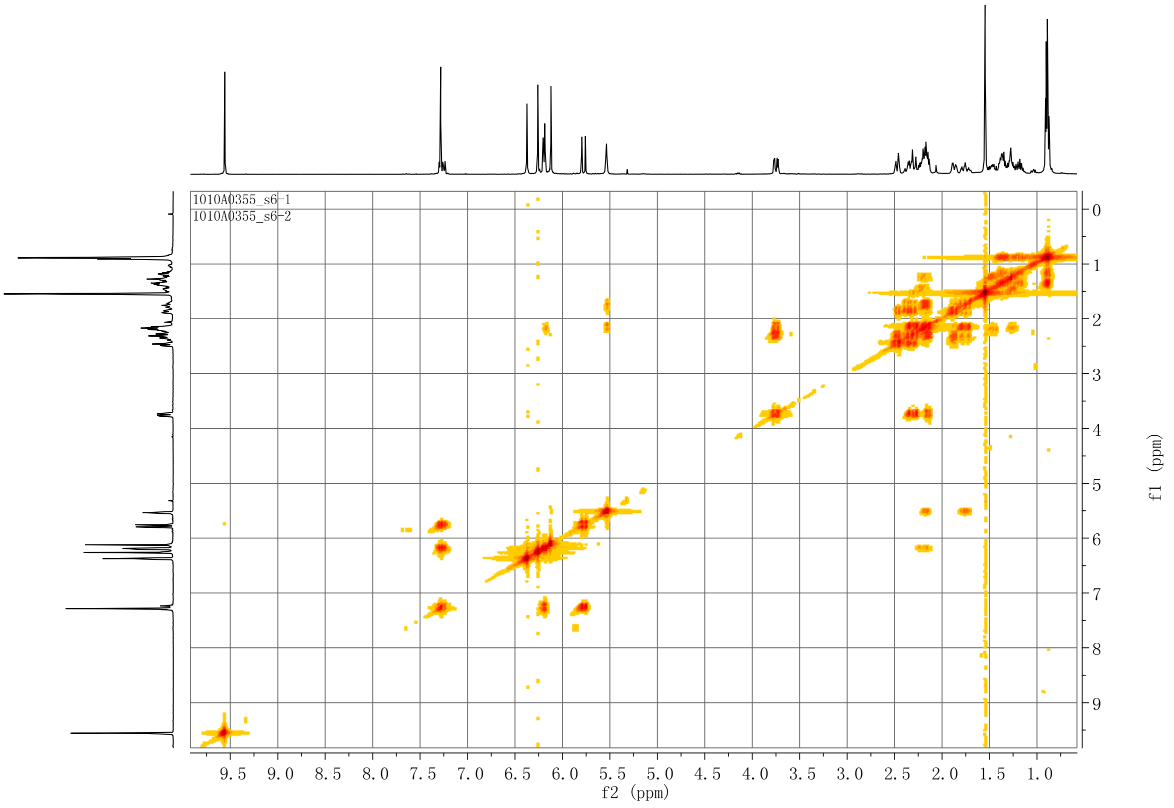

S7. HMBC spectrum of compound **1** in $CDCl_3$

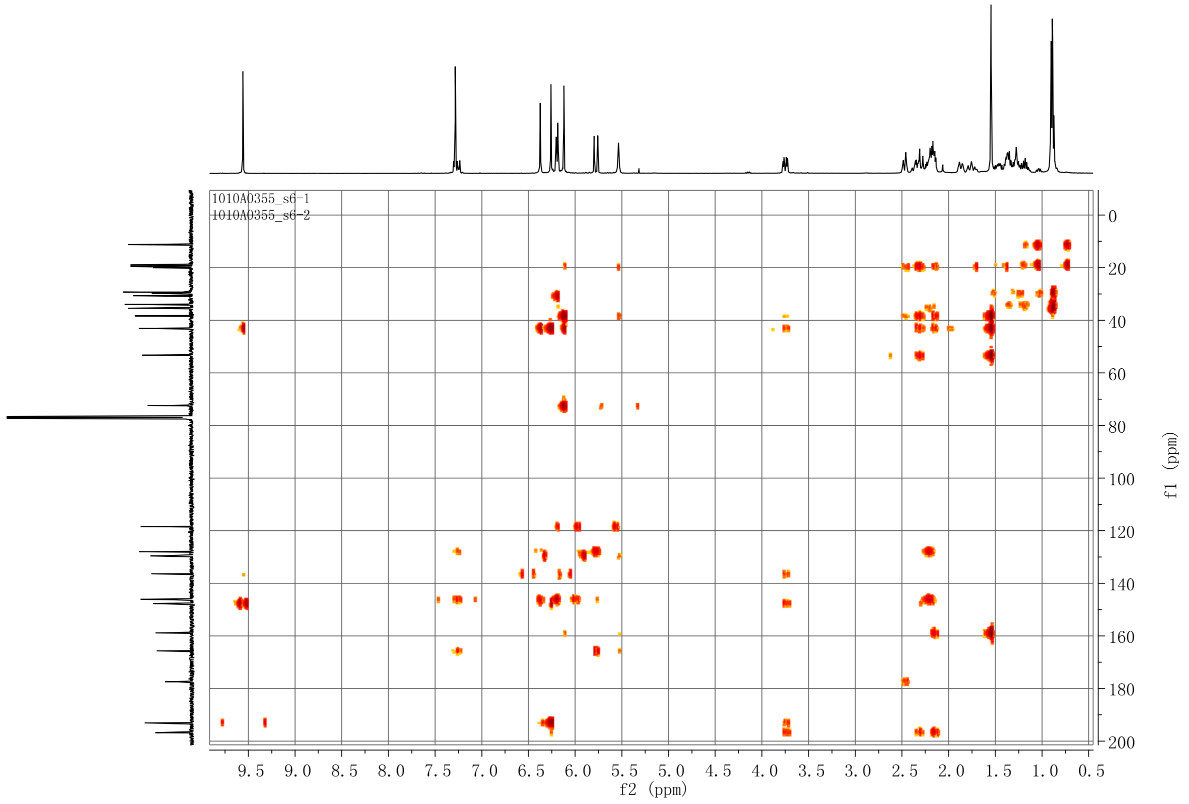

S8. NOE spectrum of compound **1** in $CDCl_3$

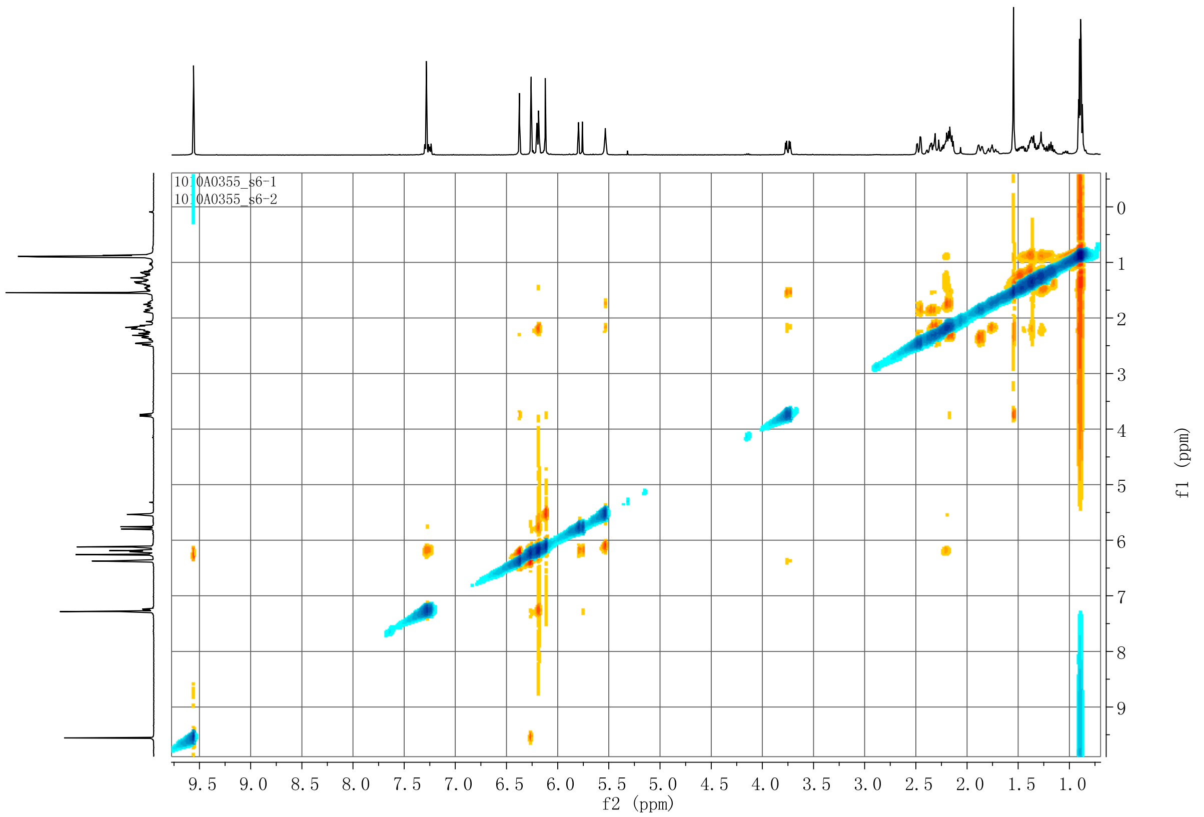

S9. EIMS of compound **2**

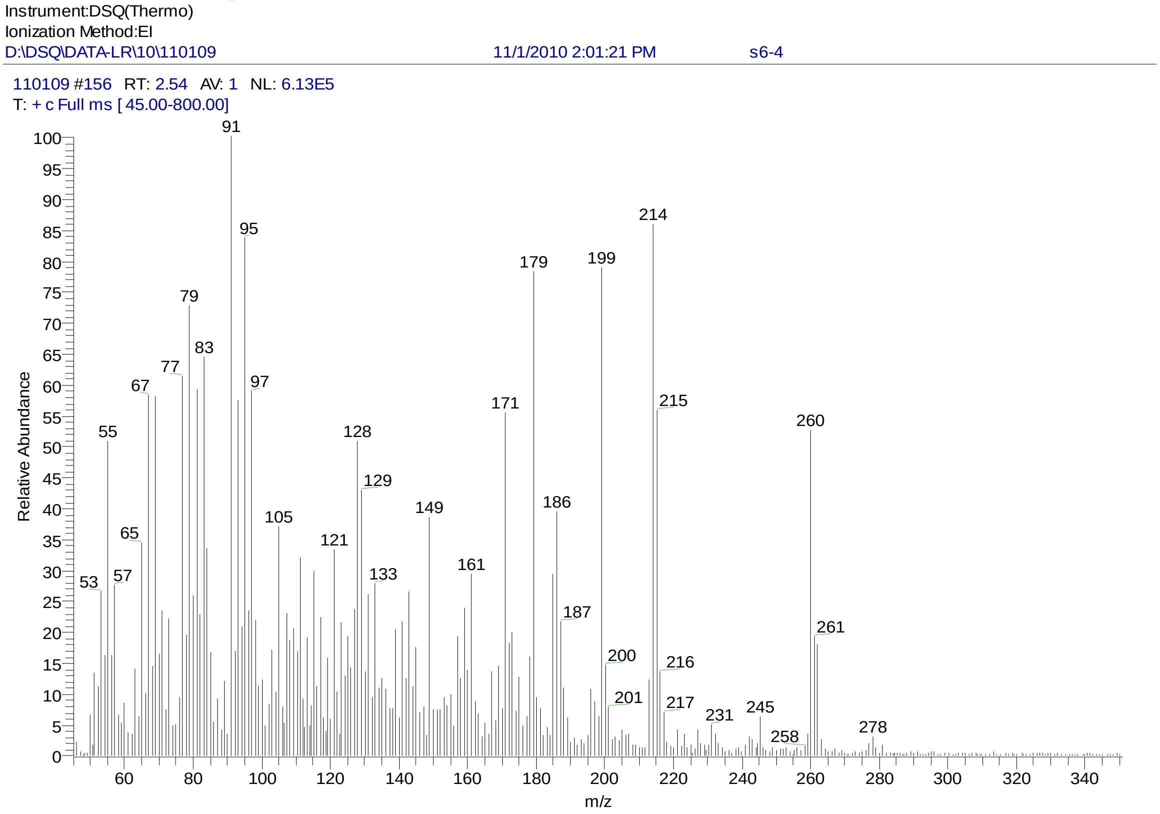

S10. ^{1}H NMR (500 MHz, $CDCl_3$) spectrum of compound **2**

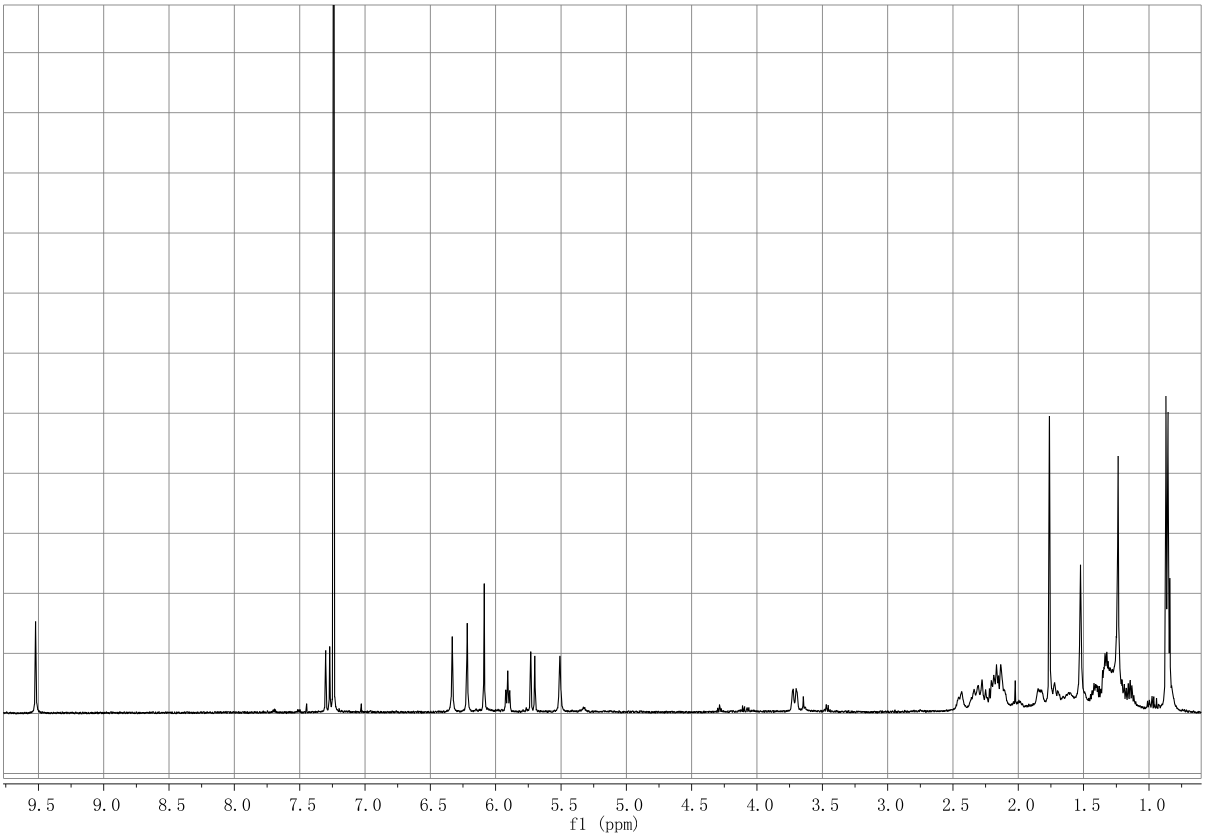

S11. ^{13}C NMR (125 MHz, $CDCl_3$) spectrum of compound **2**

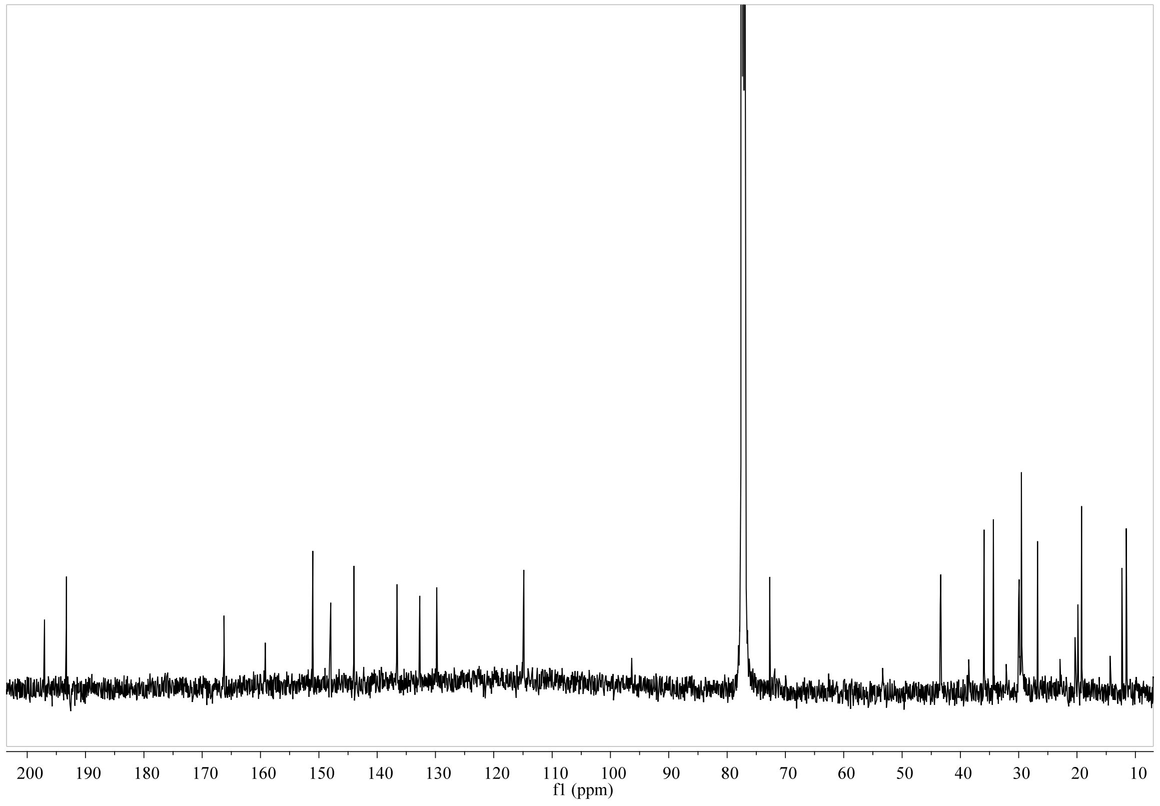

S12. HMQC spectrum of compound 2 in $CDCl_3$

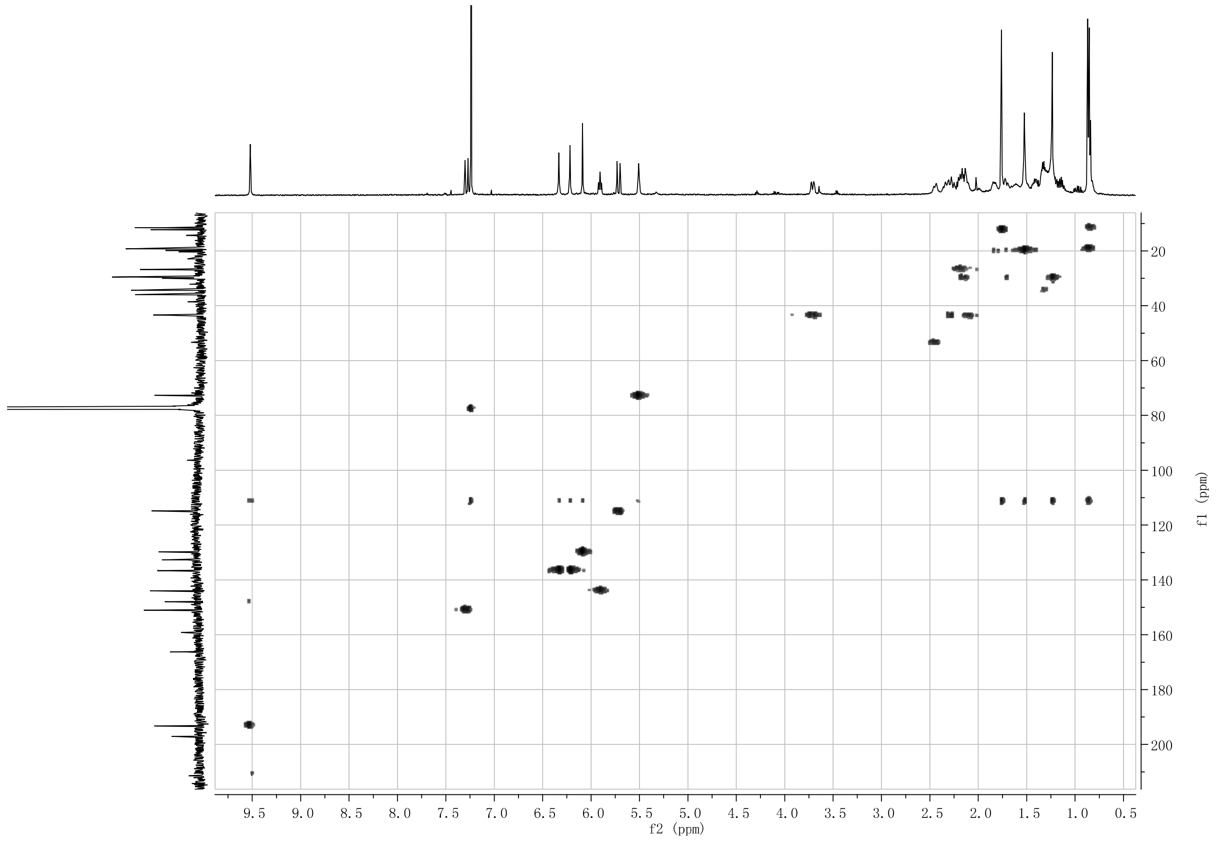

S13. H-H COSY spectrum of compound **2** in $CDCl_3$

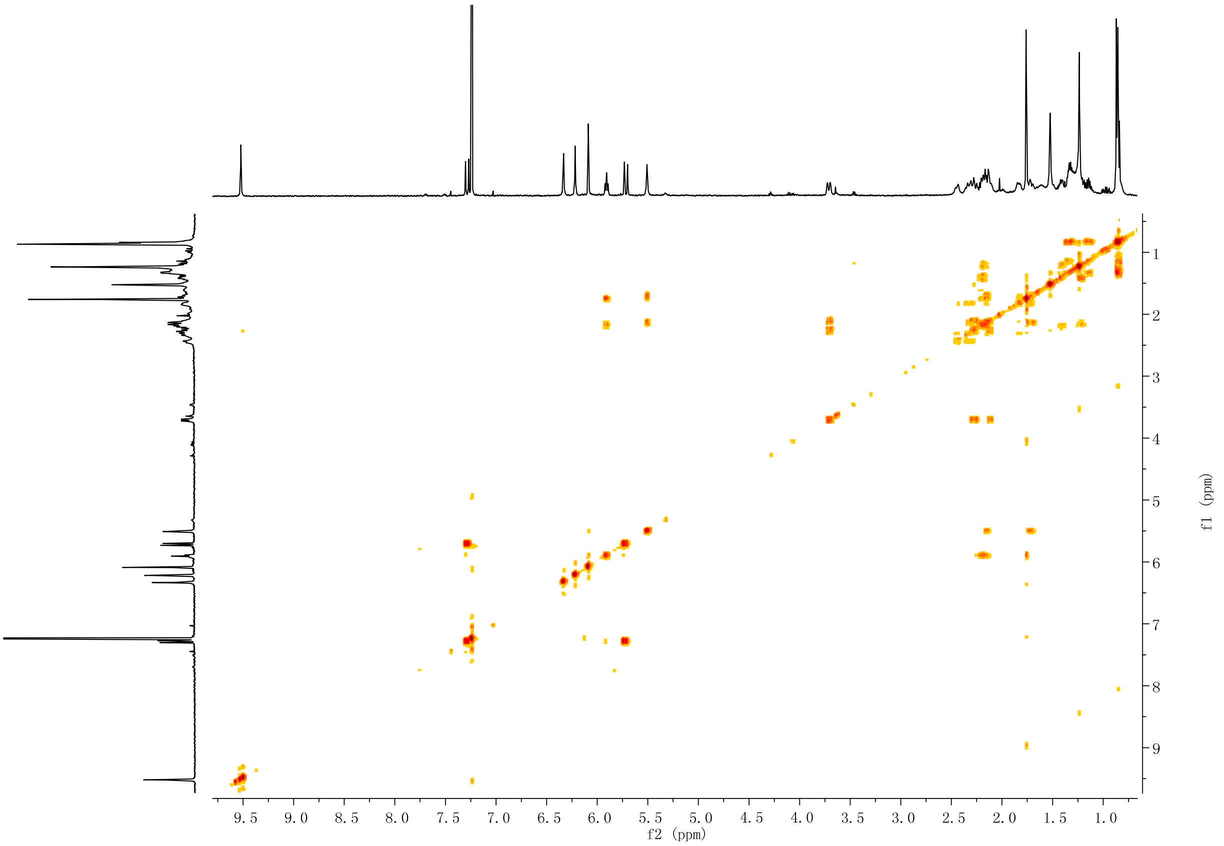

S14. HMBC spectrum of compound **2** in $CDCl_3$

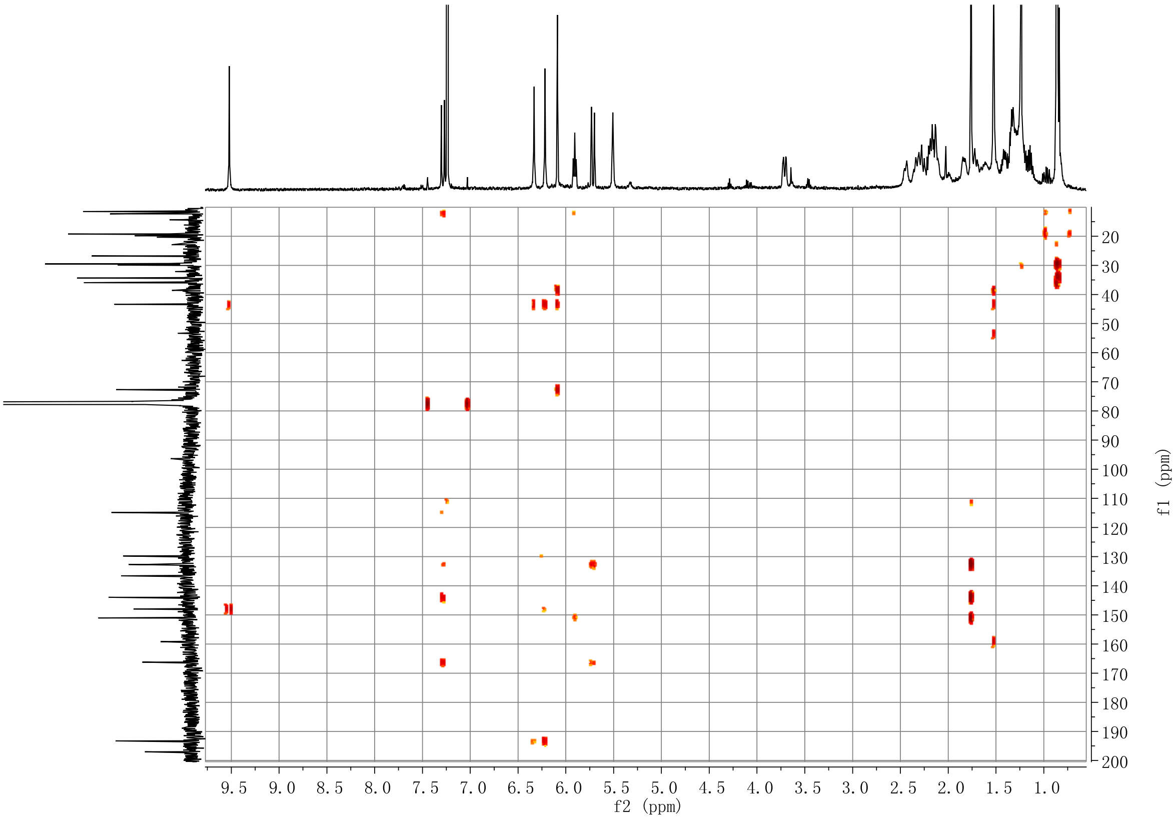

S15. NOE spectrum of compound **2** in $CDCl_3$

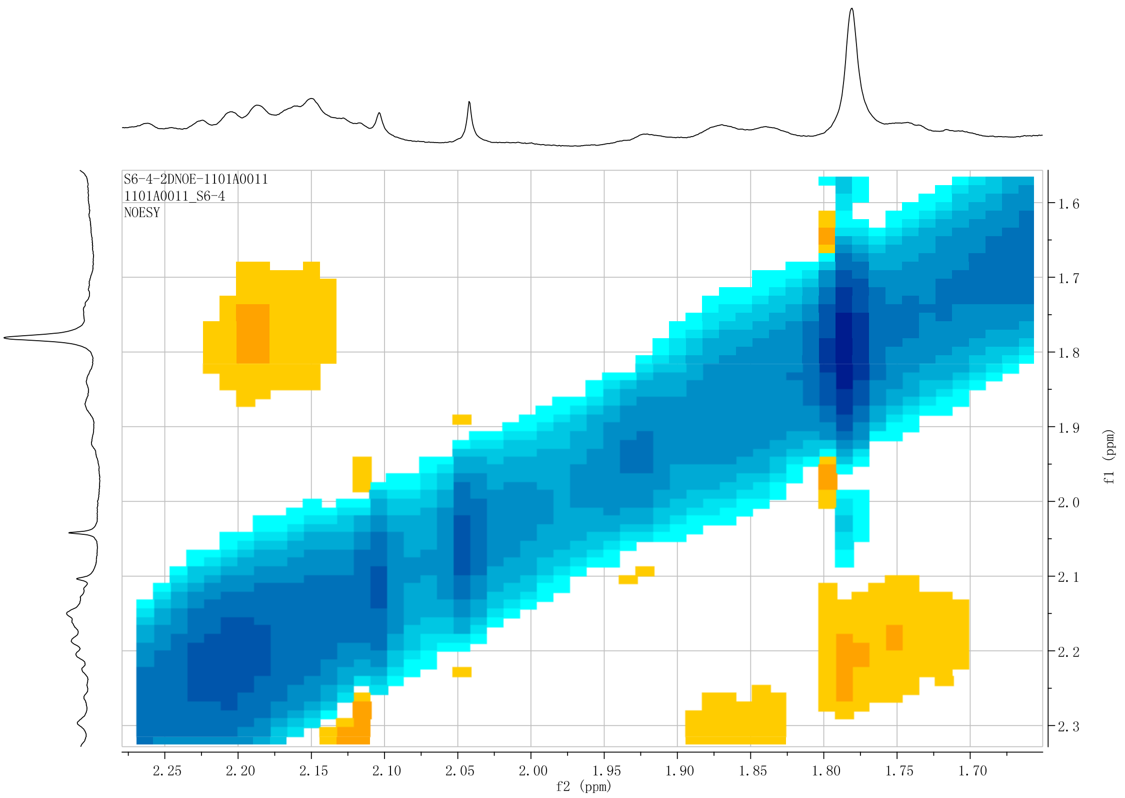

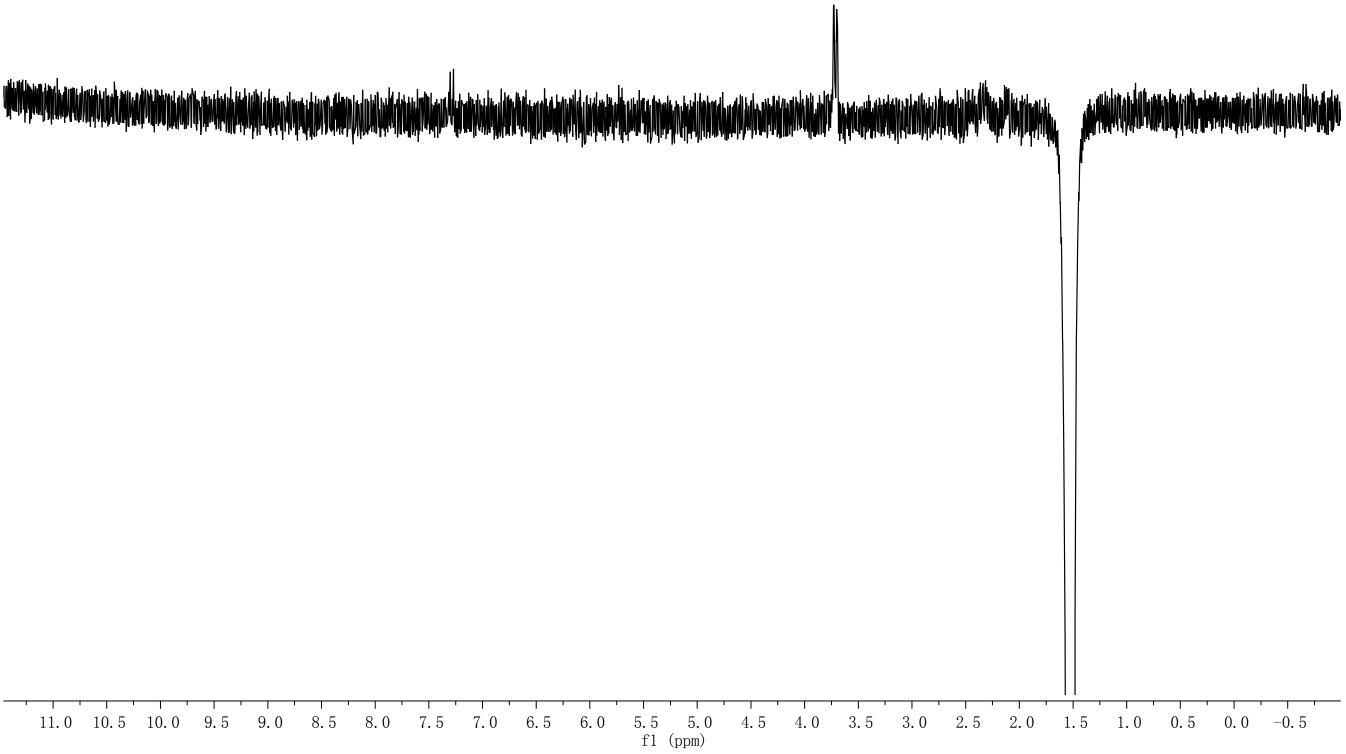

S16. EIMS of compound **3**

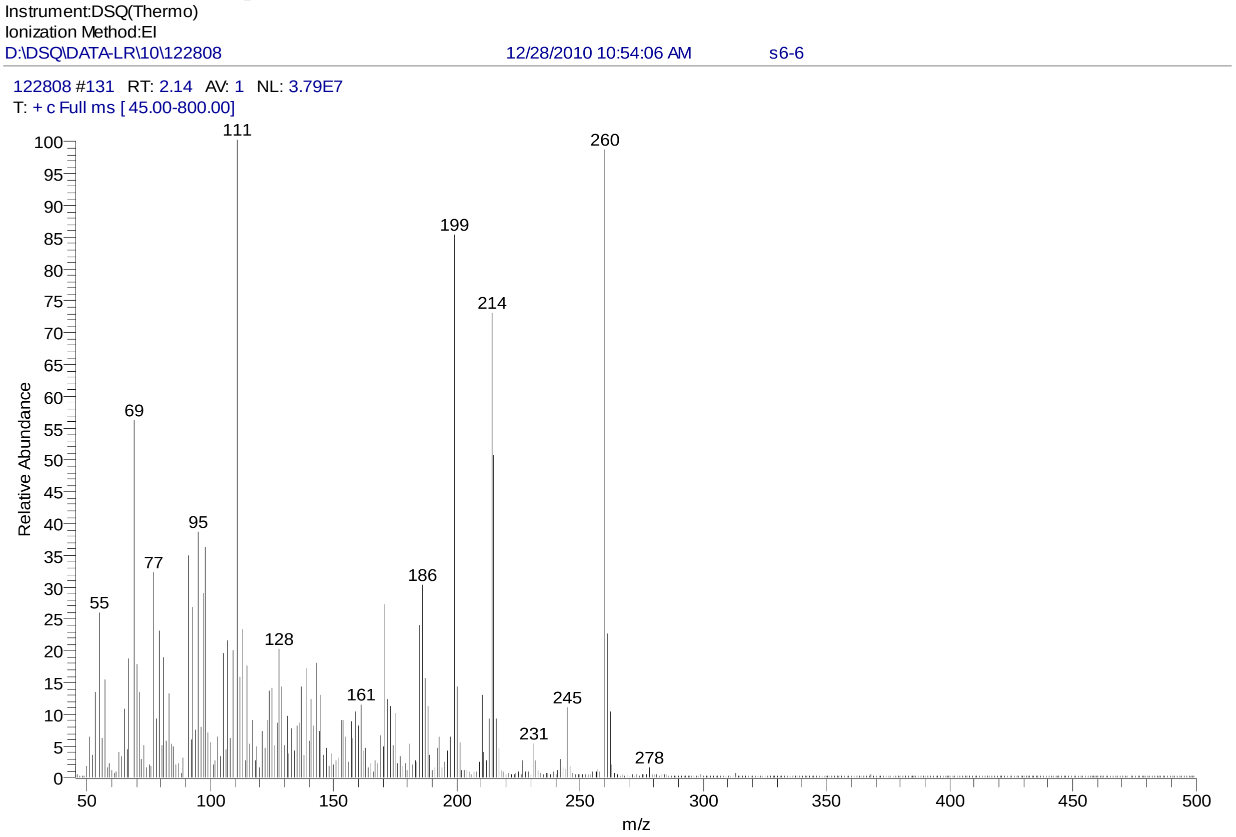

S17. ^{1}H NMR (500 MHz, $CDCl_3$) spectrum of compound **3**

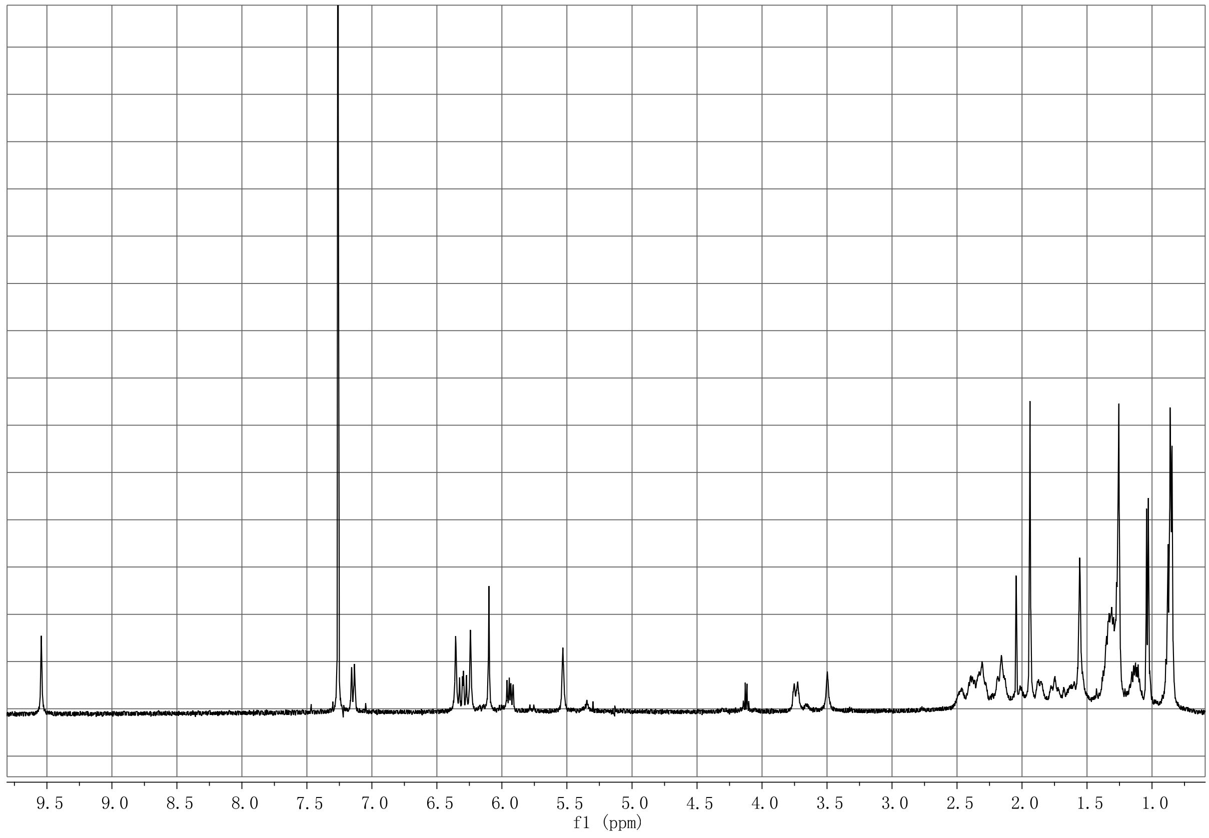

S18. ^{13}C NMR (125 MHz, $CDCl_3$) spectrum of compound **3**

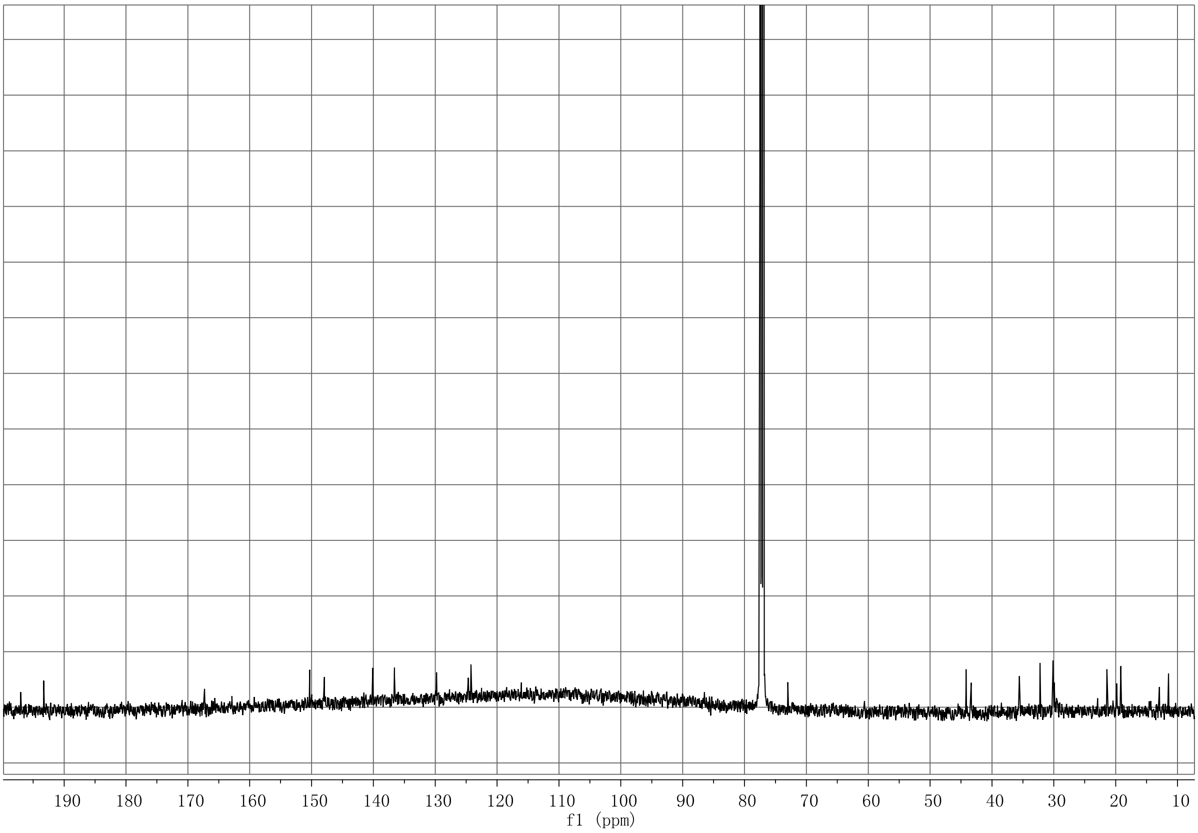

S19. HMQC spectrum of compound **3** in $CDCl_3$

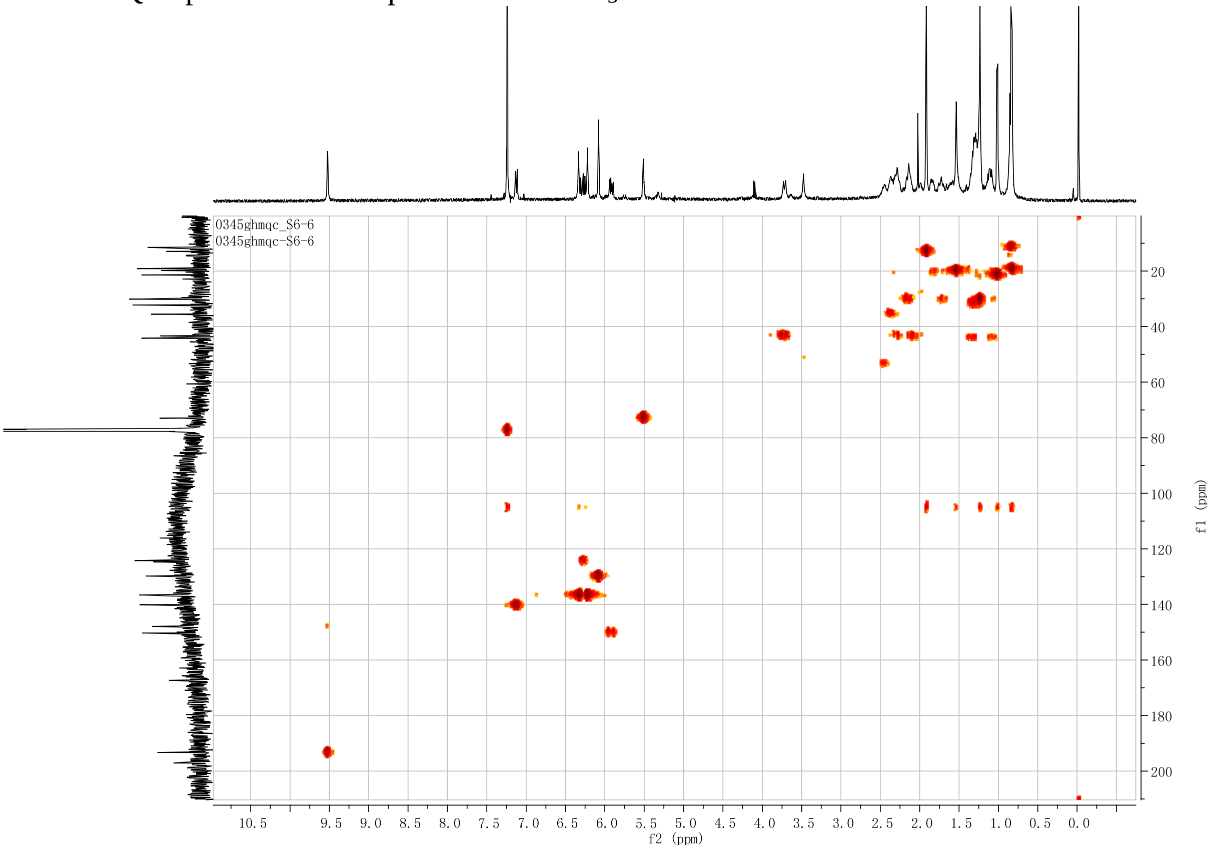

S20. H-H COSY spectrum of compound **3** in $CDCl_3$

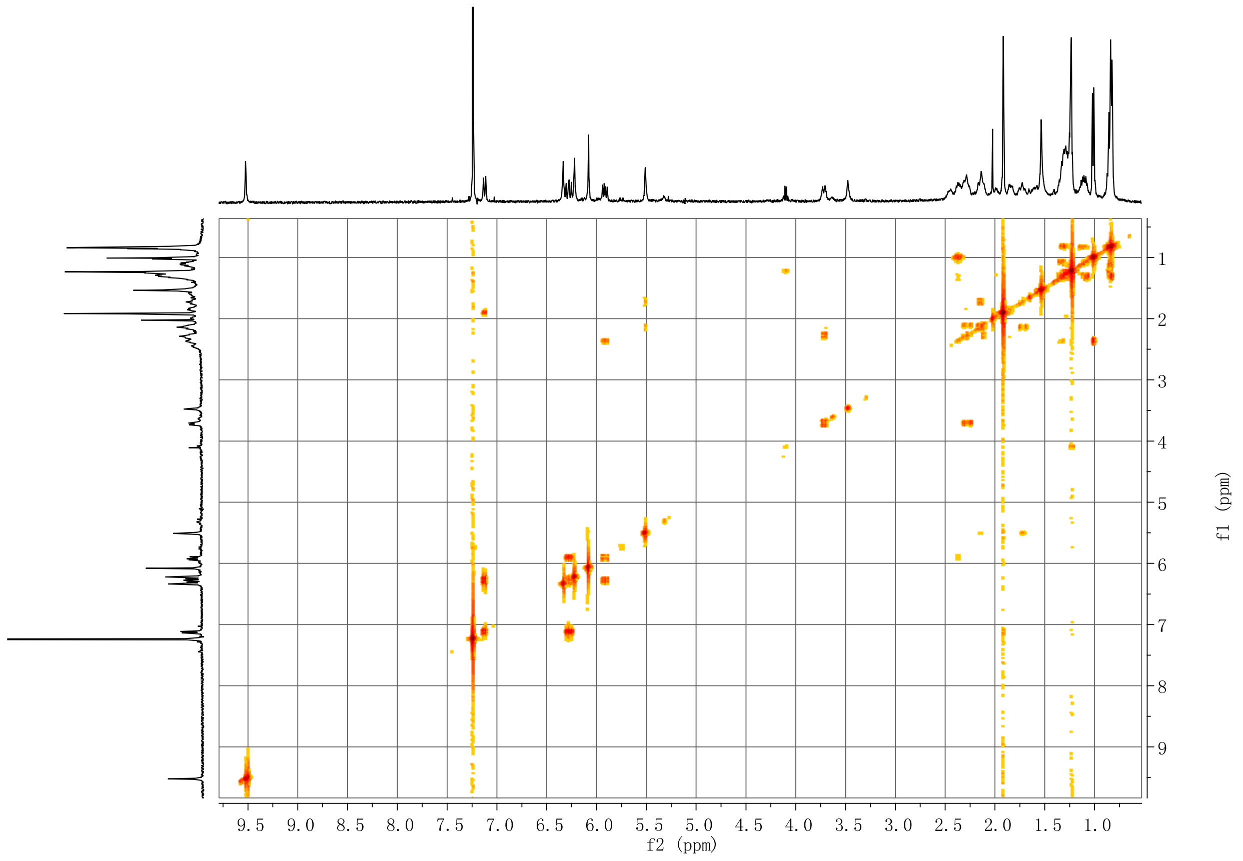

S21. HMBC spectrum of compound **3** in $CDCl_3$

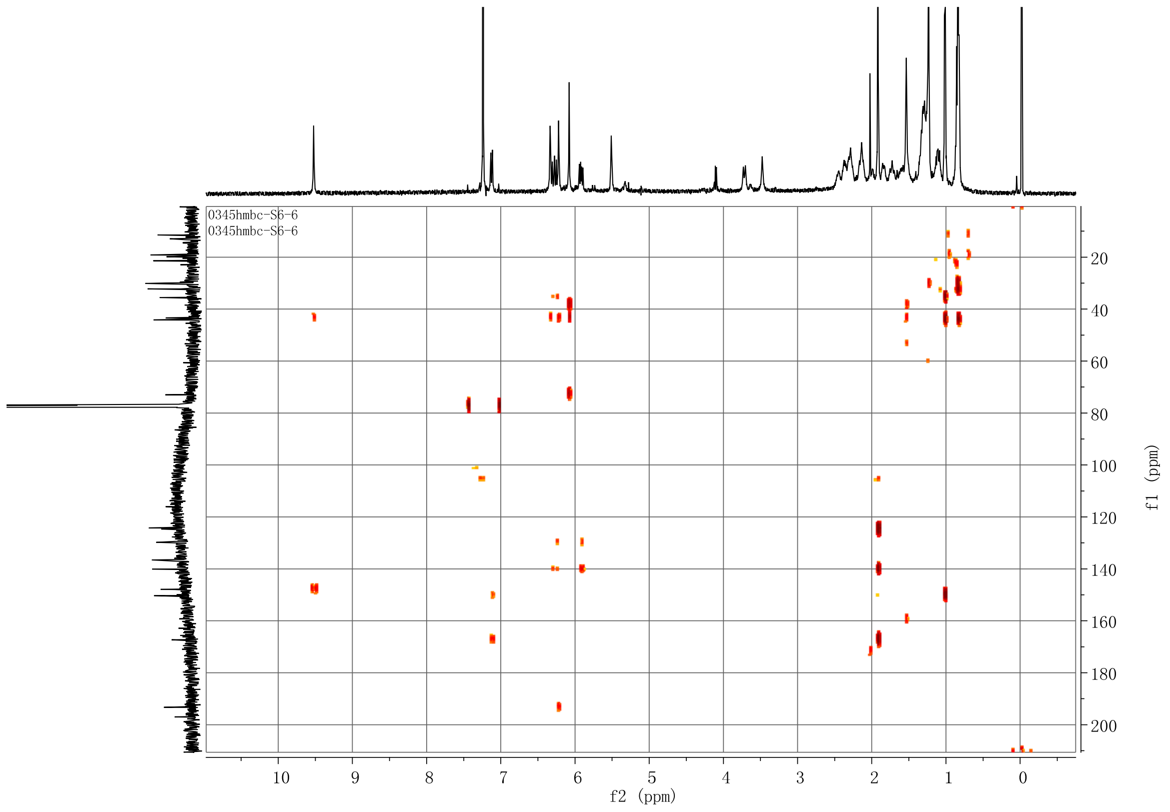

S22. NOE spectrum of compound **3** in $CDCl_3$

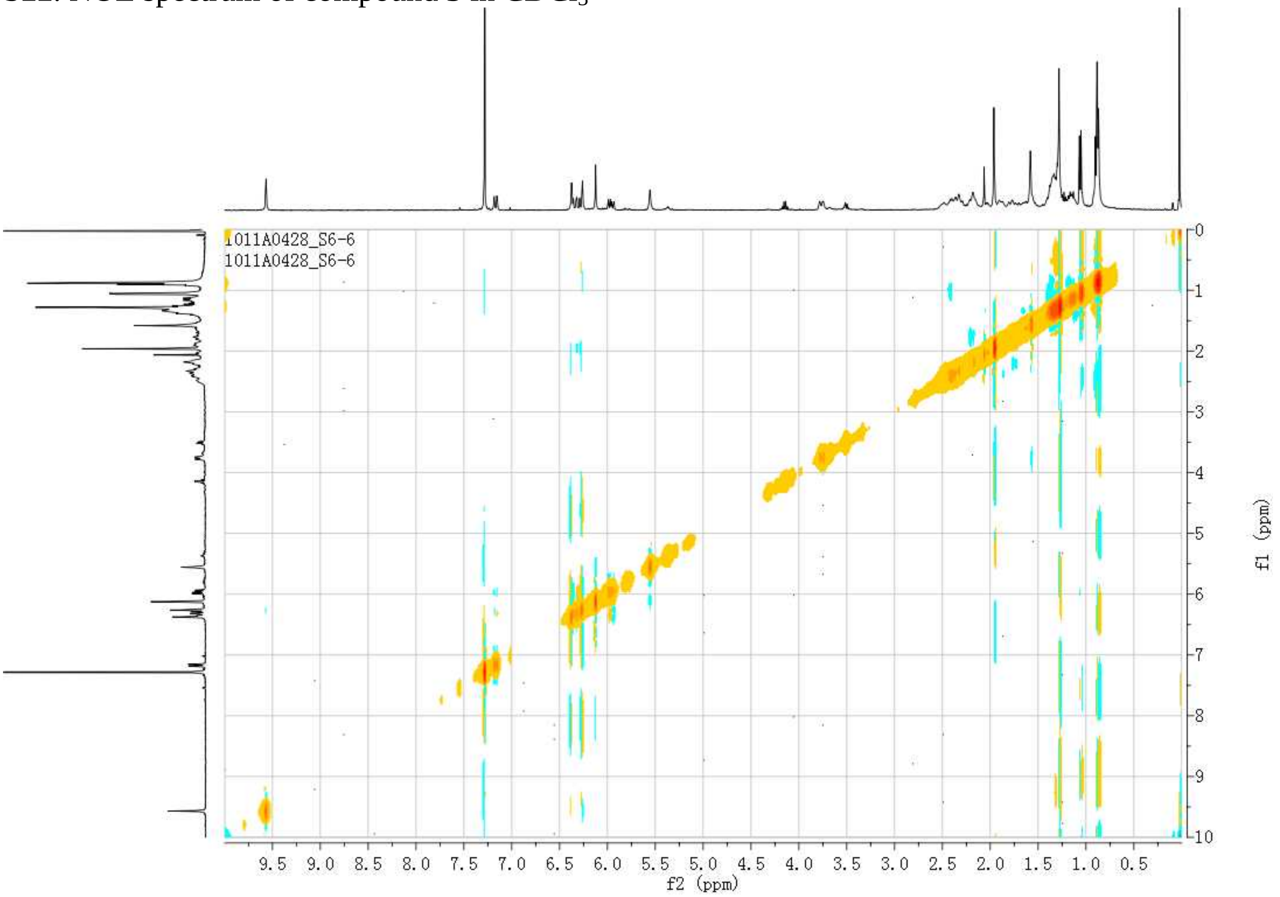

Supplement: Supplementary File 1: — PDF-Document (PDF, 614 KB) [file marinedrugs-10-00340-s001.pdf]
